# COSMIC-based mutation database enhances identification efficiency of HLA-I immunopeptidome

**DOI:** 10.1186/s12967-023-04821-0

**Published:** 2024-02-10

**Authors:** Fangzhou Wang, Zhenpeng Zhang, Mingsong Mao, Yudai Yang, Ping Xu, Shichun Lu

**Affiliations:** 1 Medical School of Chinese People’s Liberation Army (PLA), Faculty of Hepato-Pancreato-Biliary Surgery, Chinese PLA General Hospital, Institute of Hepatobiliary Surgery of Chinese PLA, Key Laboratory of Digital Hepatobiliary Surgery PLA, 28 Fuxing Road, Haidian District, Beijing, 100853 China; 2 State Key Laboratory of Proteomics, National Center for Protein Sciences (Beijing), Research Unit of Proteomics and Research and Development of New Drug of Chinese Academy of Medical Sciences, Beijing Proteome Research Center, Institute of Lifeomics, 38 Life Science Park Road, Changping District, Beijing, 102206 China; 3 School of Basic Medical Sciences, Anhui Medical University, Hefei, China; 4 Institute of Medicinal Biotechnology, Chinese Academy of Medical Sciences & Peking Union Medical College, Beijing, China; 5 School of Medicine, Guizhou University, Guiyang, China

**Keywords:** HLA immunopeptidome, Neoantigen identification, HepG2 cell, COSMIC database, Hepatocellular carcinoma

## Abstract

**Background:**

Neoantigens have emerged as a promising area of focus in tumor immunotherapy, with several established strategies aiming to enhance their identification. Human leukocyte antigen class I molecules (HLA-I), which present intracellular immunopeptides to T cells, provide an ideal source for identifying neoantigens. However, solely relying on a mutation database generated through commonly used whole exome sequencing (WES) for the identification of HLA-I immunopeptides, may result in potential neoantigens being missed due to limitations in sequencing depth and sample quality.

**Method:**

In this study, we constructed and evaluated an extended database for neoantigen identification, based on COSMIC mutation database. This study utilized mass spectrometry-based proteogenomic profiling to identify the HLA-I immunopeptidome enriched from HepG2 cell. HepG2 WES-based and the COSMIC-based mutation database were generated and utilized to identify HepG2-specific mutant immunopeptides.

**Result:**

The results demonstrated that COSMIC-based database identified 5 immunopeptides compared to only 1 mutant peptide identified by HepG2 WES-based database, indicating its effectiveness in identifying mutant immunopeptides. Furthermore, HLA-I affinity of the mutant immunopeptides was evaluated through NetMHCpan and peptide-docking modeling to validate their binding to HLA-I molecules, demonstrating the potential of mutant peptides identified by the COSMIC-based database as neoantigens.

**Conclusion:**

Utilizing the COSMIC-based mutation database is a more efficient strategy for identifying mutant peptides from HLA-I immunopeptidome without significantly increasing the false positive rate. HepG2 specific WES-based database may exclude certain mutant peptides due to WES sequencing depth or sample heterogeneity. The COSMIC-based database can effectively uncover potential neoantigens within the HLA-I immunopeptidomes.

**Supplementary Information:**

The online version contains supplementary material available at 10.1186/s12967-023-04821-0.

## Introduction

Primary liver cancer ranks as the fifth most common tumor and the second leading cause of cancer-related deaths in China. The majority of primary liver cancer cases are hepatocellular carcinoma (HCC), constitutes the majority of primary liver cancer cases, representing approximately 75–85% of all liver cancer cases [1]. Over 50% of HCC patients are diagnosed with advanced HCC, characterized by a poor prognosis and a 1-year survival rate ranging from 12 to 38% 1-year [2, 3]. Identifying effective treatment options for advanced HCC is crucial. Immune checkpoint inhibitors and antiangiogenic targeted drugs have recently emerged as first-line treatment for advanced HCC, demonstrating promising objective response rate. However, due to drug resistance, nearly half of the patients have an unsatisfactory prognosis [3, 4]. Thus, the development of efficient second-line therapies is essential. Recent reports suggest that adoptive T-cell therapy achieves promising results against various cancers, including HCC, by improving immunosuppression in the tumor microenvironment [5–7]. To prepare adoptive T-cell with high specificity and cytotoxicity against tumor cells, antigens that specifically stimulate T-cell are of high priority [8]. Increasing evidence suggests that neoantigens, characterized by tumor-specific mutant proteins or peptides with immunogenicity, are ideal antigens for T-cell activation, facilitating effective immune responses against tumors while minimizing the incidence of autoimmune reaction [9].

Conventional approaches for identifying neoantigen typically involve Next-generation sequencing or Mass spectrometry (MS) to detect somatic mutations in whole cell proteome [10–12]. These approaches, combined with in silico HLA-I binding affinity prediction, can identify neoantigen [13]. However, only a fraction of these mutant peptides is considered as neoantigens, limiting their clinical application. In comparison to previous strategies such as genomic or proteogenomic approaches, HLA-I immunopeptidome approach shows great potential in neoantigen identification directly detecting HLA-I presented peptides. This approach has been successfully used in identifying neoantigen in melanoma, non-small lung cancer and other cancers [12, 14–16], resulting in improved accuracy. Nonetheless, the identification of HLA-I immunopeptide is based on database generated from the genome, which may result in the omission of mutant peptides due to undetected low-frequency mutations and differences sample collection [17]. For instance, a study involving 8 HCC patients identified 11,266 non-synonymous single-nucleotide DNA variants, but only 1,875 amino acid mutations at the proteomic level [18]. Similarly, in another study involving whole-exome and transcriptome sequencing of 16 HCC tumor tissues and normal tissue samples, 1,039 mutations and 159 potential tumor neoantigen peptides were identified and verified by proteomics, but no corresponding HLA peptides were found from tumor tissue [19]. HCC is considered a low tumor mutational burden (TMB) cancer compared to other types of cancer [20], resulting in fewer mutations at the genomic level. Moreover, previous studies have identified a long-tail phenomenon in tumor mutation genes, leading to a high prevalence of low-frequency mutations [21]. Inadequate sequencing depth of WES poses challenges in the detection of low-frequency mutations. Due to the limitations of exome sequencing technology and cost, uneven sequencing depth is observed, resulting in insufficient coverage in SNP-intensive regions, which hinders the detection of existing variations. Consequently, WES may fail to detect mutations, especially in tumors with low frequencies [22]. These findings indicate that the number of mutations at the protein level is significantly reduced, posing challenges in identifying mutant protein or peptides recognized by the immune system [23]. This presents an increased difficulty in identifying tumor neoantigens in HCC. The Catalogue of Somatic Mutations in Cancer (COSMIC) is a comprehensive database that collects somatic mutations identified in various types of cancers, including HCC. Additionally, COSMIC provides a wealth of information on other less common genetic alterations in HCC, extending mutation database for identification of neoantigens, apart from the well-established HCC-associated genes.

This study proposes a database generation strategy to enhance the coverage of somatic mutations in HCC. The approach is based on HCC mutation data from COSMIC somatic mutation database. To evaluate the effectiveness of the strategy, we enriched and analyzed HLA-I presented peptides from HepG2 cell line using high-resolution mass spectrometry. Both COSMIC-based and HepG2 WES-based database were employed to identify potential neoantigens. Furthermore, the identified neoantigens underwent validated through HLA-I binding affinity prediction and peptide-protein docking models.

## Materials and method

### HepG2 Cell line

The HepG2 hepatocellular carcinoma cell line was obtained from the American Type Culture Collection and cultured in Dulbecco's modified Eagle's medium supplemented with 10% fetal bovine serum in 37 ℃ with 5% CO_2_.

### Western blot analysis to assess HLA-I

Samples containing HLA- I peptide complexes, including total cell lysate (TCL), flowthrough (FT), and elution fractions, were collected and separated on a 10% SDS-PAGE gel subsequently, the proteins were transferred onto a nitrocellulose membrane. The membrane was blocked with 5% defatted milk in TBST for 2 h to prevent nonspecific binding. For HLA-A/B detection, the membrane was incubated with a primary antibody against HLA-A/B (ABclonal, Hubei, China) for 2 h at room temperature. This was followed by incubation with a secondary antibody for 1 h at room temperature. Finally, signal detection was performed using a chemiluminescent substrate (Scientific, California, United States).

### HLA-I immunopeptidome enrichment and purification

To prepare the cell lysate, three biological replicates of HepG2 cells were collected and washed three times with cold phosphate-buffered saline (PBS). The cells were then lysed using a cold solution of lysis buffer consisting of 0.25% sodium deoxycholate, 0.2 mM iodoacetamide, 1 mM EDTA, and 1:200 Protease Inhibitors Cocktail (Sigma-Aldrich, Missouri, United States) in PBS. After 30 min on ice, the lysate was centrifuged at 20,000 g for 30 min at 4 ℃ to remove sediment. To enrich HLA-I peptide complexes, a house-made pan-HLA class I antibody was coupled with Protein G Sehparose beads (Cytiva, Massachusetts, United States). The beads with antibody and HepG2 cell lysate were co-incubated overnight at 4 ℃ to specifically bind the HLA-I peptide complexes. Subsequently, the captured HLA-I peptide complexes were subjected to three cold PBS washes to remove nonspecifically bound proteins or contaminants. Finally, the HLA-I peptide complexes were eluted from the beads using 0.15% Trifluoroacetic acid (TFA) in water. To purify the immunopeptidome, house-made C_18_ stage tips were prepared. The stage tips were activated by 100% acetonitrile (ACN) and 80% ACN in 0.1% TFA. followed by equilibration with 0.1% TFA in water. The HLA-I peptide complexes were loaded onto the C_18_ stage tips twice, followed by three washes 0.1% TFA in water. The purified HLA-presented peptides were eluted from the C_18_ stage tips using a solution of 30% ACN in 0.1% TFA, and subsequently dried using freeze vacuum drying equipment. To prepare for LC–MS/MS analysis, the peptide samples were reconstituted in a loading buffer containing 1% ACN in 0.1% TFA.

### LC–MS/MS analysis of HLA-I immunopeptidome

The HLA-I immunopeptidome was analyzed using an EASY-nLC 1200 instrument (Thermo Fisher Scientific, California, United States) equipped with a self-packed capillary column (75 μm i.d. × 20 cm, 1.9 μm C_18_ reversed-phase fused silica) coupled to an Orbitrap Exploris 480 (Thermo Fisher Scientific, California, United States). The gradient was comprised of an increasing Buffer B (Buffer A: 0.1% FA in water; Buffer B: 0.1% FA in 100% ACN) from 5 to 10% for 4 min, 10 to 30% for 56 min, 30 to 95% for 6 min and holding for 2 min. Full MS scans ranged from 300 to 1600 m*/z* with a resolution of 60,000. The maximum injection time was 50 ms, and the normalized automatic gain control (AGC) target was set at 300% for the remaining settings. The MS_2_ scan was configured to collect fragmentation for charge state of 2 to 6, using high-energy collision dissociation (HCD) with normalized collision energy of 27%, the resolution for MS_2_ was set at 15,000, AGC target was set at 100%, and the maximum injection time was 40 ms. A dynamic exclusion time of 15 s was applied. Theoretical retention time were generated using DeepLC (https://iomics.ugent.be/deeplc/).

### Generation of database and searching of MS raw files

Initially, the UniProt Human database (42,397 entries including isoforms) was employed in HLA immunopeptidome analysis [25]. Then, we generated a HepG2 WES-based database. The whole exome sequencing data was acquired from Depmap Portal (https://depmap.org/portal/) [26]. These acquired files were annotated by Biopython (v 1.8.1) [27], which retained only non-synonymous variants, including single nucleotide (SNV), insertions and deletions. These mutations were translated into protein sequences, which could be utilized in database search section. For the generation of COSMIC-based database, the liver and hepatocellular carcinoma were selected in the cancer browser tool in COSMIC website to generate a list of 3306 HCC samples. Then, somatic mutation information (Cosmic_GenomeScreensMutant_Tsv_v98_GRCh38) was downloaded from COSMIC (https://cancer.sanger.ac.uk/cosmic/) [28]. Somatic mutations of the HCC samples were extracted based on the HCC sample list, and nucleotide sequences were subsequently generated using the reference sequence (Genome Reference Consortium Human Build 38). Synonymous variants were removed, and the Biopython package was used to translate the nucleotide sequences into amino acid sequences to build the COSMIC-based database. However, the large size of the database meant that this process required a significant amount of resources for database searching. To improve database searching efficiency, new mutant sequences were created based on the extension of 50 amino acids (a.a) up and downstream of the altered position. If the mutation site resided within 50 amino acids from either the 3’ or 5’end, the new mutant sequences terminated as fare as possible. This approach significantly reduces the size of COSMIC-based database and improve database searching efficiency.

The database search was carried out using MSFragger (V20.0) [29] with MS raw files and UniProt Human database. MSFragger settings utilized the “non-specific HLA” workflow for HLA immunopeptidome analysis. Since the HLA-I immunopeptidome consists of proteolytically degraded proteins synthesized by the cell, no enzyme digestion was selected. A tolerance of 20 ppm was allowed in both the MS and MS_2_ search modes. A false discovery rate (FDR) of < 1% was set for peptide-spectrum match, and no protein FDR filter was applied. The workflow assumed that cysteines were not alkylated, and cysteinylation was specified as a variable modification. The HepG2 WES-based and COSMIC-based database were also used for the database search in MSFragger, utilizing the same settings.

### HLA-I typing and HLA binding prediction

HLA typing of HepG2 was obtained from a previous study [30]. Additionally, HLA typing was performed using arcasHLA with the HepG2 transcriptome sequence from our previous work [31, 32]. The confirmed HLA typing results for HepG2 cell line were used in the subsequent analysis. Only peptides consisting of 8–14 a.a were selected for HLA binding prediction. The peptides were clustered into groups based on sequence similarities using Gibbscluster-2.0 with default parameters [33]. Briefly, Kullback–Leibler Divergence in each group was calculated in Gibbscluster, and group with the highest Kullback–Leibler Divergence was selected for the most obvious difference between peptide segments. HLA-I binding prediction was performed using NetMHCpan-4.1 (https://services.healthtech.dtu.dk/services/NetMHCpan-4.1/) with HLA subtype HLA-A0201, HLA-A2402, HLA-B3514, HLA-B5101, HLA-C0401, HLA-C1602. The threshold was 0.5 and 2 for strong and weak binder respectively, and NetMHCpan built-in evaluation data sets were used for binding prediction. Binding prediction results were visualized using R studio (build 524) with ggplot2 package, and the artwork is created with BioRender.com.

### Peptides-protein docking modeling

Mutant peptides identified from the HepG2 WES-based and COSMIC-based database searches underwent several filtering steps based on spectrum, length, and HLA binding results. Only mutant peptides with a length of 8–14 a.a and strong HLA-I binding were selected for peptides-protein docking modeling. The structure model of HLA-A0201 (1DUZ) and HLA-A2402 (5HGA) were downloaded from RCSB Protein Data Bank [34]. HPepDock 2.0 (http://huanglab.phys.hust.edu.cn/hpepdock/), a computationally efficient protein-peptide prediction model, was used for docking modeling [35]. The prediction results were automatically evaluated by HPepDock according to the interface Root Mean Square Deviation from the native structure. The peptide-protein docking model and molecular surface hydrophobicity were subsequently analyzed and visualized using ChimeraX (v 1.6.1) [36].

## Results

### Identification and characteristics of HLA-I immunopeptidome in HepG2 cell line

**Fig. 1:**
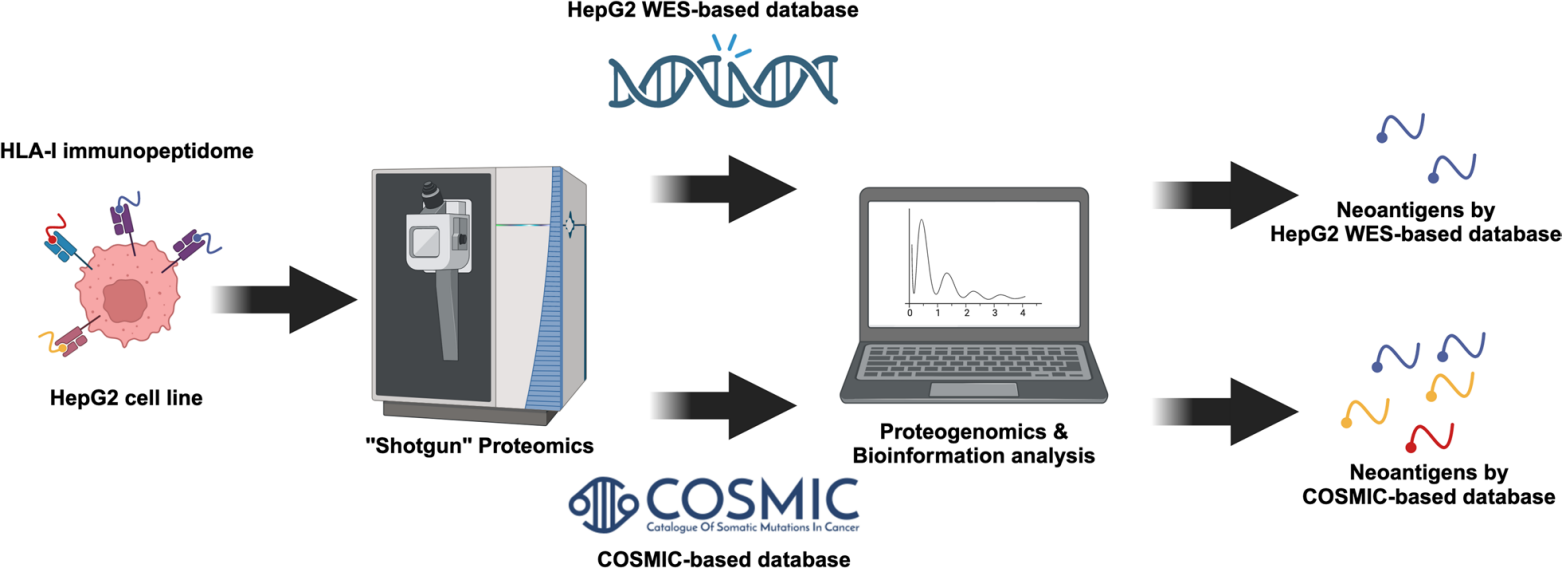
Workflow of neoantigens identification from HepG2 HLA-I immunopeptidome. The HepG2 cell HLA-I immunopeptide complex was enriched using the W6/32 antibody. The immunopeptides were then separated from the HLA-I immunopeptide complex using a C_18_ column and identified through LC-MS/MS analysis. A HepG2 WES-based and COSMIC-based mutation database were utilized to identify potential neoantigens from the immunopeptidomes. Finally, these neoantigen candidates were assessed using bioinformatics tools to confirm their affinity to the HLA-I molecule.

**Fig. 2:**
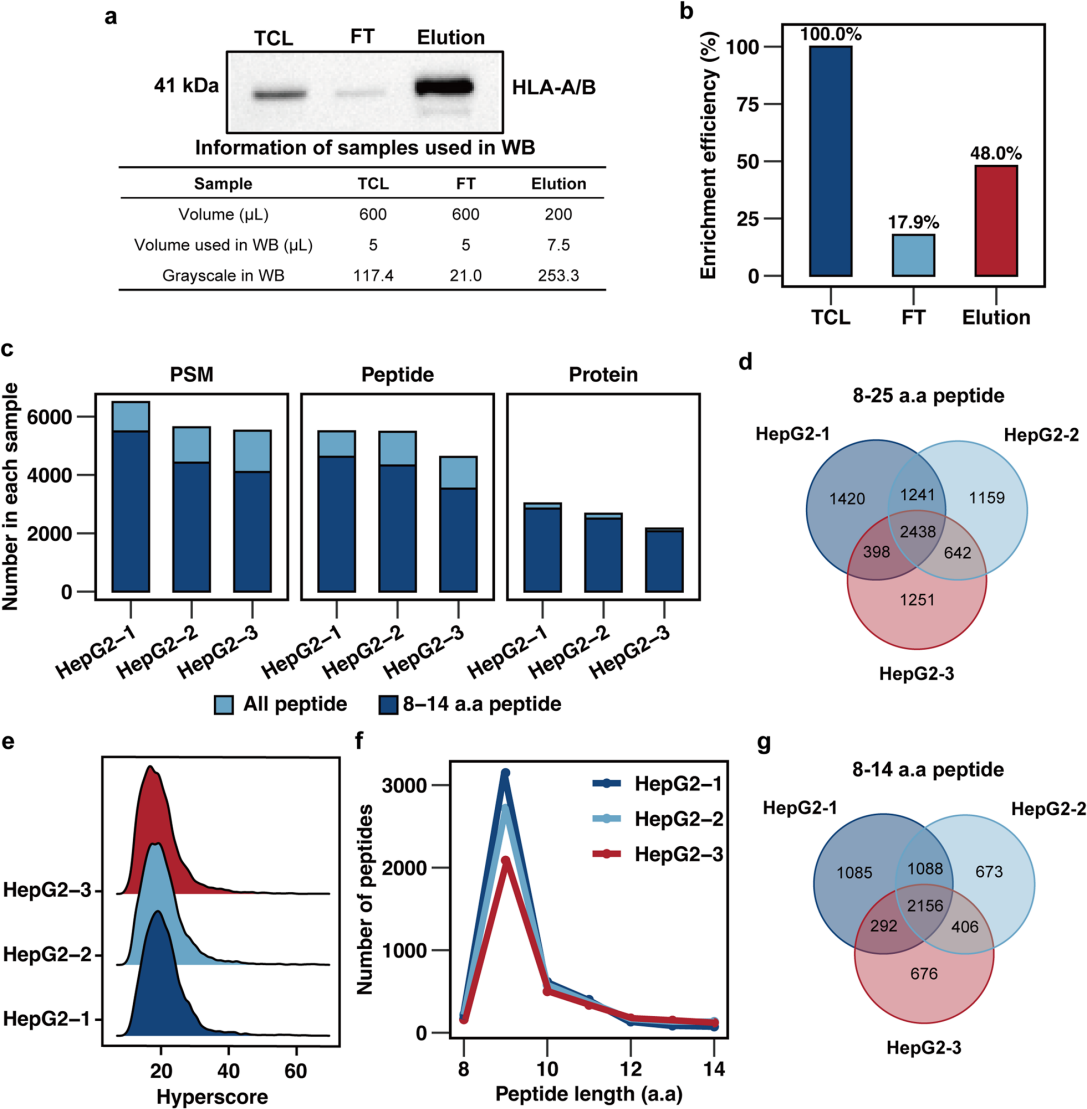
HLA-I immunopeptides identified from HepG2 by IP-MS. **A**. Immunoblot showing HLA A/B protein levels in total cell lysate (TCL), flowthrough (FT) and elution. Table shows volumes used in western blotting analysis and corresponding grayscale value. **B**. HLA-A/B protein enrichment efficiency based on immunoblot result. **C**. Comparison of the matched MS spectra and identified peptides as well as proteins. **D**. Overlap of identified peptides for 3 biological replicates from HepG2 samples using Uniprot Human database. **E**. Distribution of Hyperscore of immunopeptides identified from 3 repeated HepG2 samples. **F**. Length distribution of with 8-14 a.a peptides identified from 3 repeated HepG2 samples. **G**. Overlap of 8-14 a.a identified peptides for three biological replicates from HepG2 cells using Uniprot Human database.

The process of identifying neoantigens is illustrated (Fig. 1). Immunoprecipitation successfully enriched HLA-I peptide complexes from HepG2 cell lysate. To evaluate the enrichment efficiency, we compared the HLA A/B signals in TCL, FT and elution by western blotting, quantifying them using grayscale (Fig. 2a, Additional file 1: Fig. S1a). Western blot analysis revealed that the HLA A/B signal in the FT was significantly lower compared to the TCL, while a strong signal was observed in the elution. Greyscale quantification demonstrated successfully enrichment and elution of nearly 50% of HLA A/B and eluted from the TCL (Fig. 2b). Subsequently, a total of 8549 peptides were identified through LC–MS/MS analysis. Of these, approximately 74.6% of them (*n* = 6,376) being 8–14 a.a in length, which corresponds to the length characteristics of HLA-I immunopeptidome ( Additional file 1: Table S1). In three biological replicates, we identified 4537, 3900 and 3481 8–14 a.a peptide sequences, respectively (Fig. 2c). Venn diagrams demonstrated commonly identified peptides comprised the highest portion, demonstrating consistency in the enrichment and identification of HLA-I immunopeptidome (Fig. 2d). Furthermore, Hyperscores, used for evaluating the quality of spectra by comparing observed spectra to theoretical ones generated by MSFragger [29], were compared between HLA-I immunopeptidome from three replicates, indicating consistency in the mass spectrum quality (Fig. 2e, Additional file 1). The theoretical reaction time (RT) exhibits a strong correlation with the observed RT (Additional file 1 Fig. S1b; Additional file 2). Peptides with lengths ranging from 8 to 14 a.a exhibited a distribution pattern, with the majority consisting of 9 a.a peptides (Fig. 2f, g and Additional file 1: Fig. S1c).

### Discover HepG2 specific mutant peptide using HepG2 WES-based database

**Fig. 3:**
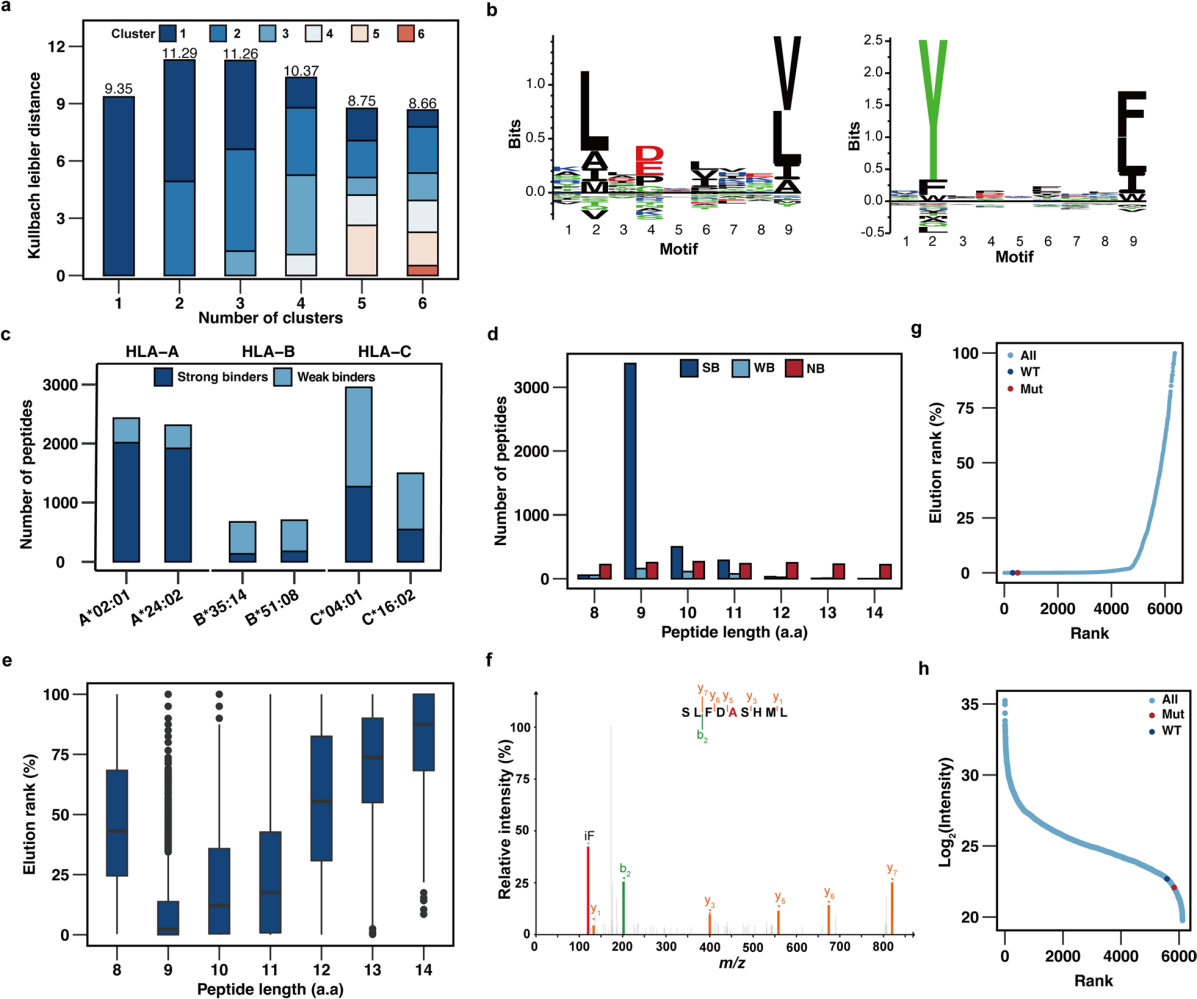
Mutant peptides identified from HepG2 HLA-I immunopeptidome using HepG2 WES-based database. **A**. Kulbach leibier distance of different cluster numbers calculated by Gibbs cluster tool. **B**. MS-identified clustered to reveal the main binding motifs. **C**. NetMHCpan prediction of each MS-identified peptides assigned to different HLA alleles using HepG2 WES-based database. **D**. Distribution of NetMHCpan prediction strong, weak and non-binders according to the length of peptides. **E**. Lowest NetMHCpan predicted for binding which were rank and normalized to percentage value. **F**. MS2 spectra of a mutant HLA-I immunopeptide “SLFDASHML”. **G**. Elution rank distribution of mutant peptide and corresponding wild-type peptide in HLA-I immunopeptidome. **H**. Intensities distribution of mutant peptide and corresponding wild-type peptide in HLA-I immunopeptidome.

Based on the satisfied data quality of the HepG2 HLA-I immunopeptidome, we utilized HepG2 WES-based database to identify potential neoantigens from HLA-I immunopeptidome (Additional file 3). The Gibbs clustering approach was used to analyze the anchor residues of the eluted peptides. The results revealed primary binding motifs that primarily clustered in two groups (Fig. 3a, b, Additional file 4). Subsequent analysis was performed using NetMHCpan 4.1 with default settings. Peptides were categorized as having strong binding when the percentage rank was less than 0.5%, and weak binding was assigned to those within the percentage rank range of 0.5 to 2.0%. The results indicated that 66.8% of 8–14 a.a peptides were predicted to bind to HLA-I molecules. A preference for binding was observed across different HLA alleles, with HLA-A0201 (n = 2014) and HLA-A2402 (n = 1918) exhibiting particularly strong preference (Fig. 3c). The distribution of amino acid species at the P2 (Second amino acid) and PΩ (Last amino acid) positions in the Gibbs clustered peptides corresponds with the distribution observed in the NetMHCpan reference database (Additional file 1: Fig. S2d, e).

These findings are consistent with the results obtained from Gibbs clustering, and indicating that the binding motif clusters within HLA-A0201 and HLA-A2402. Additionally, there was a length distribution among strong binders, and it was found that 9 a.a peptides were the most frequently observed (Fig. 3d), which is consistent with the length distribution of HLA-I immunopeptides reported in previous studies[12, 14, 37]. Furthermore, 9 a.a peptides were predicted to have a lower elution percentage rank, indicating a stronger binding ability to the HLA-I molecules (Fig. 3e). However, there was no significant difference in peptide intensity observed among peptides of different lengths (Additional file 1: Fig. S2a). Our study identified only 1 mutant peptide, derived from mutation of thymine to cytosine at position 242 in AMT, using the HepG2 WES-based database (Table 1, Fig. 3f), indicating the limited effectiveness of personalized-specific databases in identifying mutant immunopeptidomes. Similar findings were reported in a previous study [16]. Notably, the HLA-I immunopeptidomes of HepG2 contained both the wild type peptide “SLFDVSHML” and the mutant peptide “SLFDASHML”. Although the mutant peptide and wild-type peptide had low intensities, their predicted HLA-I affinity was higher compared to other peptides (Fig. 3g, h).

**Table 1:** List of mutant peptides identified by HepG2 WES-based database

No	Peptide	Hyperscore	Peptide length	Nucleic variant	Amino variant	Protein	Binding level	HLA allele	Elution rank %
1	SLFDASHML	**11.98**	**9**	**c.242 T > C**	p.V81A	AMT	Strong	HLA-A*02:01	**0.003**
2	SLFDVSHML	**15.81**	**9**	**WT**	WT	AMT	Strong	HLA-A*02:01	**0.005**

### Generation of COSMIC-based database

**Fig. 4:**
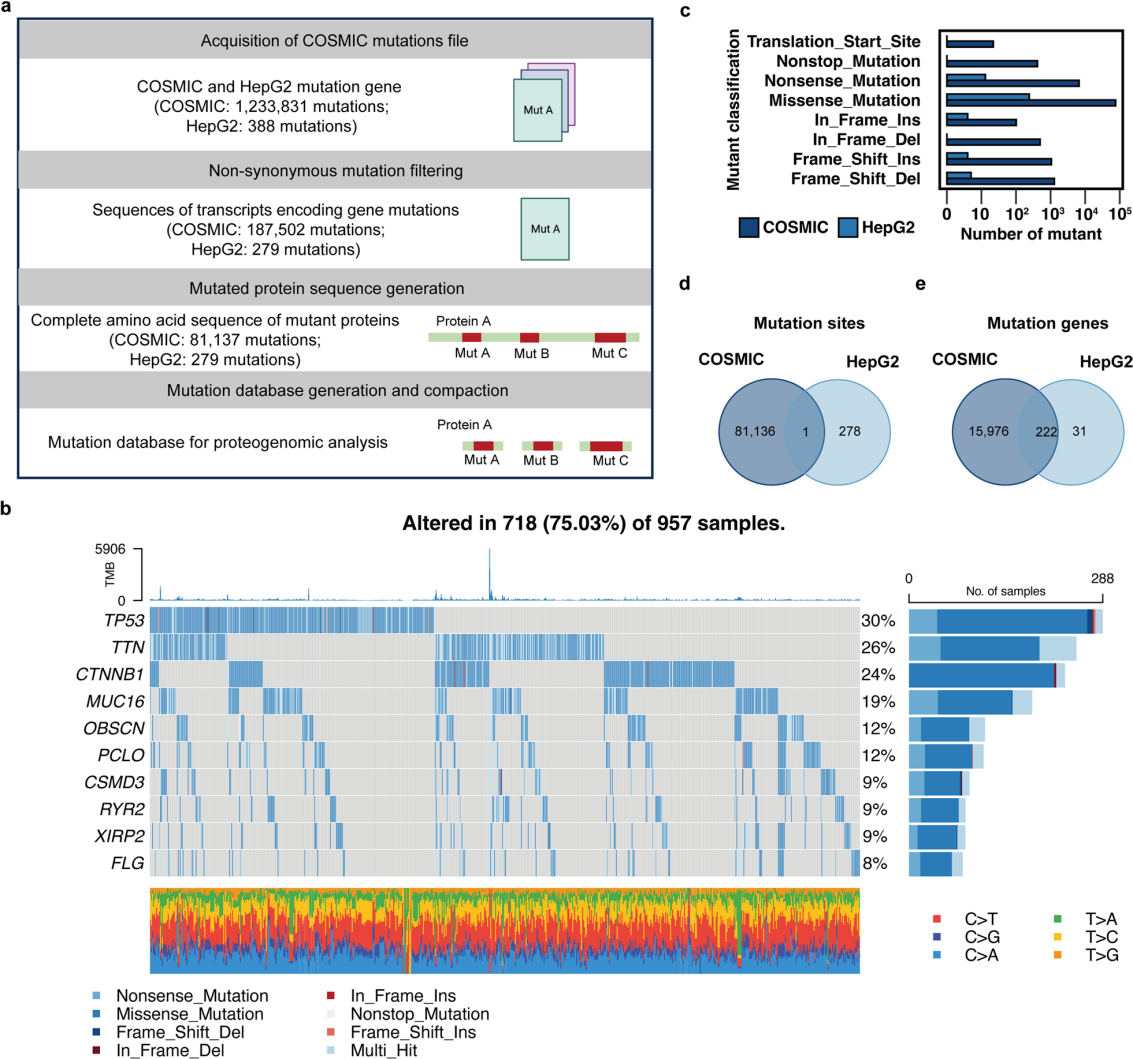
Generation COSMIC-based database**.** **A**. Workflow for the generation of COSMIC-based proteogenomic mutation database. **B**. The genomic landscape and mutational signatures in COSMIC hepatocellular carcinoma somatic mutation database; **C**. Comparison of mutated proteins distributed in different types of mutation in HepG2 (n = 279) and COSMIC-based database (n = 81,137). **D**. Venn diagram of mutation site from HepG2 and COSMIC-based database. **E**. Venn diagram of mutated genes from HepG2 and COSMIC-based database

The construction workflow for the mutation database was summarized into four parts (Fig. 4a). The genes sequences of mutations were extracted from COMSIC genomic database, which consisted of 1,233,831 mutations, and HepG2 WES database, which contained 388 mutations. Non-synonymous mutations lead to changes in the amino acid sequence. Therefore, further filtering was conducted to remove synonymous and redundant mutations. As a result, 279 mutations were identified in the HepG2 WES-based database, and 81,137 mutations were identified in the COSMIC-based database (Additional file 5). Among the 957 samples in the COSMIC-based database, TP53 was the most frequently mutated gene, followed by TTN and CTNNB1 (Fig. 4b). Notably, most of the samples exhibited a low TMB, although a few individuals displayed exceptionally high TMB. These results are consistent with previous findings that HCC has a relatively lower TMB compared to other types of tumors [19, 20]. The distribution of mutant classes in the HepG2 WES-based and COSMIC-based database was similar, with missense mutations being the most common, followed by nonsense mutations (Fig. 4c and Table 2). The proportions of other mutation types, including nonstop mutations, insertions, and deletions, were similar in the COSMIC-based database. Only 1 mutation was found in both databases, while 222 mutated genes were commonly identified (Fig. 4d, e). To conduct subsequent MS data searches, a COSMIC-based database was constructed by incorporating these filtered somatic mutations from both COSMIC and HepG2 WES into the UniProt Human database.

**Table 2:** Statistic of mutant classification in HepG2-based and COSMIC-based database

Mutant classification	HepG2-based database	COSMIC-based database
Translation start site	**0**	**21**
Nonstop mutation	**0**	**415**
Nonsense mutation	**12**	**6640**
Missense mutation	**240**	**75,883**
In frame insertion	**3**	**100**
In frame deletion	**0**	**497**
Frameshift insertion	**3**	**1052**
Frameshift deletion	**4**	**1278**

### Evaluation of HepG2 WES-based and COSMIC-based database

**Fig. 5:**
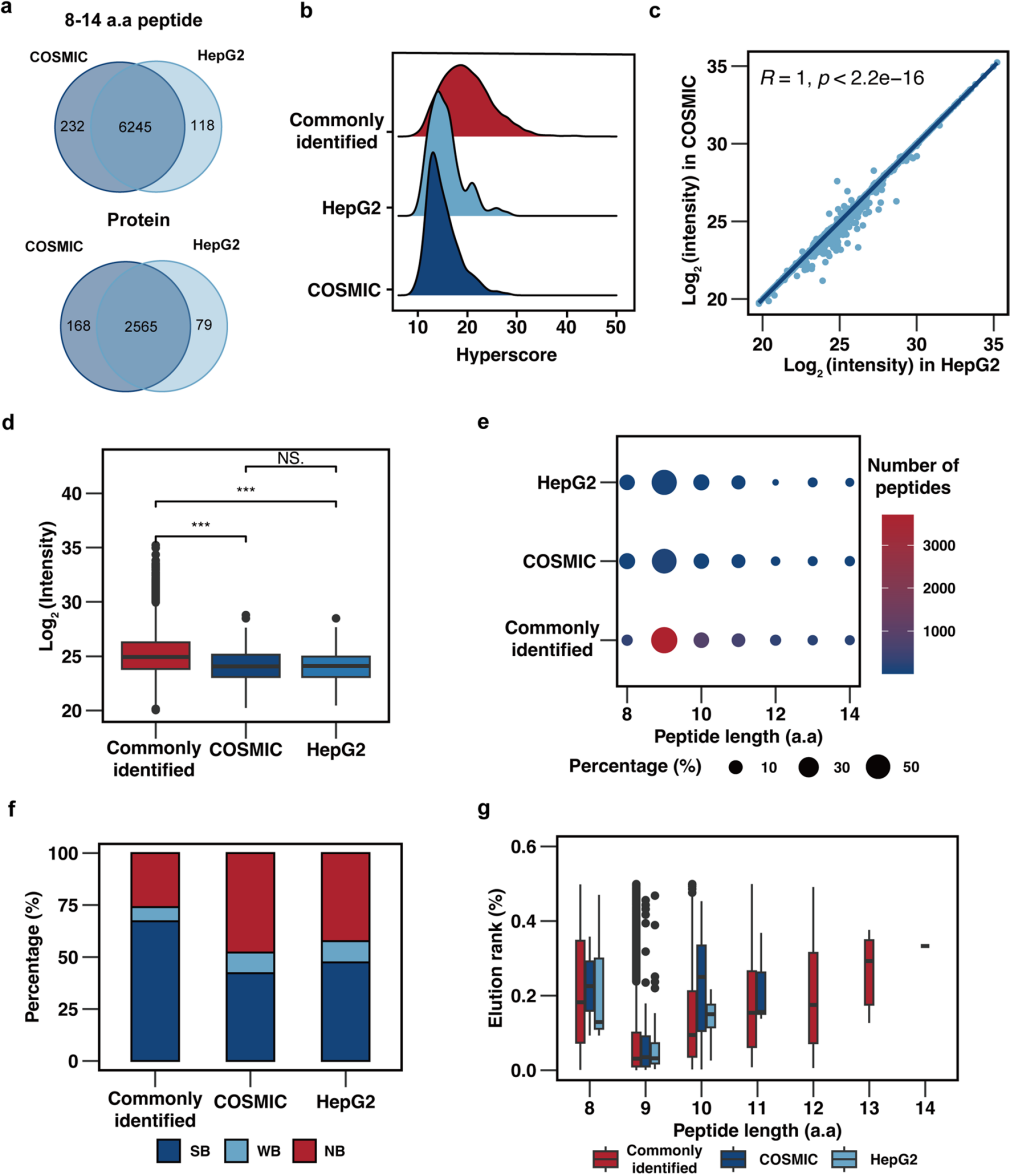
Comparison of HLA-I immunopeptides identification using HepG2 WES-based and COSMIC-based database**.** **A**. Venn diagram of identified HLA-I immunopeptides (upper panel) or proteins (lower panel) against either HepG2 WES-based or COSMIC-based database. **B**. Hyperscore distribution of commonly identified and uniquely identified HLA-I immunopeptides. **C**. Scatter plot intensity for the commonly identified peptides. **D**. Intensity comparison of commonly identified and uniquely identified HLA-I immunopeptides. **E**. Length distribution of commonly identified and uniquely identified HLA-I immunopeptides. **F**. Number of binders of identified HLA-I immunopeptides predicted by netMHCpan. **G**. Lowest netMHCpan predicted and ranked for the identified HLA-I strong-binding peptides

To evaluate the impact of HepG2 WES-based and COSMIC-based database on database search result, a comparison was made between the outcomes of the two mutation databases. Venn diagrams showed that a majority of immunopeptides (*n* = 6245) were commonly identified by both databases, resulting in a total of 2565 shared proteins (Fig. 5a, Additional file 6). The quality of unique identified peptides by each mutation database was initially evaluated (Fig. 5b). Comparative analysis revealed that, in comparison to the commonly identified peptides, the majority of unique identified peptides from either mutation database exhibited lower hyperscores, indicating lower spectrum quality. Nonetheless, a subset of spectra exhibited hyperscores higher than the average hyperscore of the commonly identified counterparts, suggesting that the use of mutation databases enables the discovery of unique peptides with high quality. Further investigation was conducted on the unique peptides identified by both databases. Incomplete product ion coverage was observed as a prevalent scenario, resulting in spectra matching to different peptides. manual inspection of the MS_2_ spectra revealed that 41 peptides, comprising 11.71% of all unique peptides, had equal-weight amino acids or combinations (Additional file 1: Fig. S3a). Furthermore, a quantitative evaluation demonstrated a strong correlation between the peptide intensities obtained from the two search results (Fig. 5c).

We observed that unique identified peptides from both mutations databases had statistically lower intensities compared to their commonly identified counterparts. However, there was no significant difference between in the intensities of unique identified peptides (Fig. 5d). Furthermore, a comparison of the length distribution patterns for commonly and uniquely identified peptides revealed that both groups exhibited a similar distribution, with the majority of peptides having a length of 9 a.a (Fig. 5e). These results imply that the unique identified peptides are highly likely to be immunopeptides. To evaluate the their immunoaffinity, we utilized NetMHCpan to predict their binding affinity. The analysis revealed that the proportion of strong binding peptides was lower in the unique identified peptides compared to their counterparts, although strong binders still accounted for nearly 50% (Fig. 5f). Additionally, the elution percentage rank distribution based on peptide length supported this conclusion, as both commonly and uniquely identified peptides demonstrated similar trends. Specifically, 9 a.a peptides showed the highest elution percentage rank among strong binders (Fig. 5g). This pattern was also evident in the immunopeptides identified using UniProt database, providing further evidence that the majority of the unique identified peptides were immunopeptides.

### Discover HepG2 specific mutant peptide using COSMIC-based database

During the evaluation of the HepG2 WES-based and COSMIC-based database for the identification of HLA-I immunopeptidome, we excluded peptides with "isoleucine" to "leucine" mutations, as these cannot be distinguished by mass spectrometry. As a result, we identified 16 mutant peptides (Table 3). Although both mutation databases include other mutant classes of mutations, such as nonsense mutations, insertions, and deletions, all of the mutant peptides identified in our study harbored SNVs instead of other mutation class. In contrast, the COSMIC-based identified 16 mutant peptides, including the one discovered using the HepG2 WES-based database. These results indicate that the COSMIC-based database is more efficient for the identification of the HLA-I immunopeptidome.

**Table 3:** List of mutant peptides identified by COSMIC-based database

No	Peptide	Origin	Hyperscore	Length	Nucleic variant	Amino variant	Protein	Binding level	HLA allele	Elution rank %
1	RYSEYTEEF	COSMIC	**22.11**	**9**	**c.1795G > A**	p.A599T	MTMR6	Strong	HLA-A*24:02	**0.003**
2	RMPEAAPRV	COSMIC	**11.81**	**9**	**c.215C > G**	p.P72R	TP53	Strong	HLA-A*02:01	**0.084**
3	SLFDASHML	HepG2 COSMIC	**11.98**	**9**	**c.242 T > C**	p.V81A	AMT	Strong	HLA-A*02:01	**0.007**
4	TVLSSRPVV	COSMIC	**13.06**	**9**	**c.1574 T > C**	p.I525T	ITGAL	Weak	HLA-B*51:08	**1.487**
5	LSWHLPLLI	COSMIC	**18.14**	**9**	**c.26G > A**	p.R9H	IL2RB	Weak	HLA-C*16:02	**1.029**
6	LNDLIVALS	COSMIC	**12.87**	**9**	**c.1668C > A**	p.F556L	NVL	None	–	–
7	KAYGSYEELAKDPN	COSMIC	**11.19**	**14**	**c.197G > A**	p.S66N	DHDH	None	–	–
8	DEAQNLTRD	COSMIC	**12.61**	**9**	**c.2177G > A**	p.G726D	DDX54	None	–	–
9	HGELLEVNL	COSMIC	**14.11**	**9**	**c.219C > A**	p.D73E	PCDHA13	None	–	–
10	LFLDAIHLT	COSMIC	**14.69**	**9**	**c.2255C > T**	p.P752L	BBX	None	–	–
11	DLLLVPTAGL	COSMIC	**18.93**	**10**	**c.346 T > G**	p.Y116D	PROM2	None	–	–
12	GTLLSGAVGSLLL	COSMIC	**19.86**	**13**	**c.508A > T**	p.T170S	SLC17A9	None	–	–
13	HMLIDLHFR	COSMIC	**12.86**	**9**	**c.559A > T**	p.M187L	FMR1	None	–	–
14	QVQLLQQQ	COSMIC	**12.12**	**8**	**c.593A > T**	p.Q198L	TFAP4	None	–	–
15	DSNRNLDLDSIIA	COSMIC	**23.42**	**13**	**c.914A > G**	p.N305S	KRT79	None	–	–
16	QVQIGTHSPP	COSMIC	**12.92**	**10**	**c.2125G > A**	p.A709T	PHEX	None	–	–

**Fig. 6:**
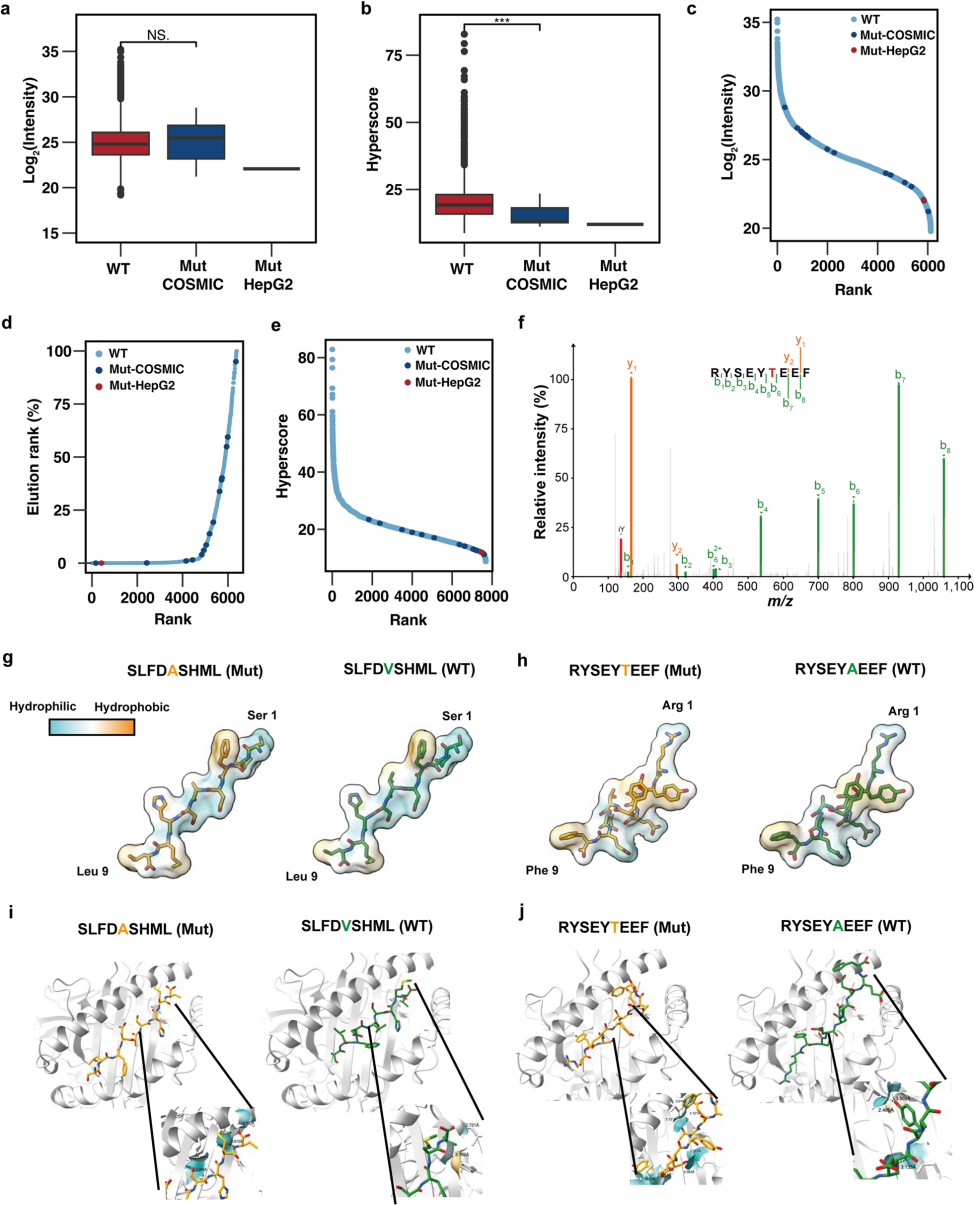
Evaluation of mutant peptides identified from HLA-I immunopeptides using COSMIC-based database**.** **A**. Intensity comparison of total peptides and mutant peptides identified HLA-I immunopeptides. **B**. Hyperscore comparison of total peptides and mutant peptides identified HLA-I immunopeptides. **C**. Intensity distribution of all of the identified HLA-I immunopeptides as well as mutant peptides. **D**. Hyperscore distribution of all of the identified HLA immunopeptides as well as mutant peptides. **E**. HLA-I affinity distribution of all of the identified HLA immunopeptides as well as mutant peptides. **F**. MS2 spectra of a mutant peptide “RYSEYTEEF”. **G**. Simulated structures of mutant peptide “SLFDASHML” and wild type peptide “SLFDVSHML” predicted by Kyte-Doolittle method. Color represents the hydrophobicity of the molecular surface. **H**. Simulated structures of mutant peptide “RYSEYTEEF” and wild type peptide “RYSEYTAEF”. Colour represents hydrophobicity of the molecular surface.** I**. Structures of mutant peptides “SLFDASHML” and “SLFDVSHML” binding groove of HLA*A02:01 predicted by HPEPDOCK. Hydrogen bond link and atomic distance between peptide and HLA. **J**. Structures of mutant peptides “RYSEYTEEF” and “RYSEYTAEF” binding groove of HLA*A24:02 predicted by HPEPDOCK. Hydrogen bond link and atomic distance between peptide and HLA-I molecule

NetMHCpan predicted binding of at least one HLA allele for 5 of the mutant peptides (Table 4). Further investigation of the mutation peptides exclusively found in HCC revealed the presence of aminomethyltransferase (AMT) _p.V81A_, integrin alpha-L (ITGAL) _p.I525T_, and interleukin-2 receptor subunit beta (IL2RB) _p.R9H_. Interestingly, myotubularin-related protein 6 (MTMR6) _p.A599T_ was also identified in large intestine cancer, while cellular tumor antigen p53 (TP53) _p.P72R_ was confirmed in multiple cancers affecting the bone, skin, meninges, and large intestine (Additional file 1: Fig. S3b). These findings suggests that peptides derived from mutations occurring in multiple cancers could potentially serve as neoantigens, stimulating tumor cytotoxic T-cells against a variety of cancers. Next, we compared the spectral quality and intensities of mutant peptides to those of wild-type peptides in immunopeptidomes. The intensities of mutant peptides showed no significant difference compared to those of normal peptides (Fig. 6a, Additional file 7). Specifically, the intensities of mutant peptides were evenly distributed across the overall peptide (Fig. 6c), with the majority of mutant peptides falling within a linear range. However, the hyperscores of mutant peptides were significantly lower than those of normal peptides, indicating poorer spectrum quality for the mutant peptides (Fig. 6b). The distribution of hyperscores revealed that mutant peptides were mainly concentrated in the sub-average region (Fig. 6d). A similar distribution pattern was observed in HLA-I affinity of mutant peptides (Fig. 6e).To assess the spectrum quality of mutant peptides, a manual inspection was performed, which revealed a high product ion coverage, particularly at the mutant amino acid, in spectra with high hyperscores. This finding increased our confidence in the accuracy of the mutant peptides (Fig. 6f). Conversely, it was also observed that some spectra with high quality were ranked with low hyperscores (Additional file 1: Fig. S4a, b). However, the majority of mutant peptides had lower spectrum quality than wild-type peptides. Common observations in spectra of low-quality peptides included incomplete product ion coverage and low relative intensity of product ions, which can result in low hyperscores. Furthermore, incomplete product ion coverage may lead to single or multiple amino acid mismatches, thus resulting in wild-type peptides being mistaken as mutant peptides. This discrepancy also explains the lower proportion of binders among mutant peptides compared to wild-type peptides. For further analysis, 3 mutant peptides were selected based on their affinity and satisfactory spectrum quality.

**Table 4:** Summaries of HLA affinity, molecular docking energy score

No	Peptide	Type	HLA affinity (nM)	HLA Allele	HLA template	Docking energy score
1	SLFDASHML	Mutant	**4.93**	HLA-A0201	1DUZ	**− 232.927**
2	SLFDVSHML	Wild type	**4.63**	HLA-A0201	1DUZ	**− 256.297**
3	RYSEYTEEF	Mutant	**9.98**	HLA-A2402	5HGA	**− 260.960**
4	RYSEYAEEF	Wild type	**13.32**	HLA-A2402	5HGA	**− 213.461**

We performed molecular structure prediction and peptide-protein docking modeling to confirm the binding affinity of mutant peptides. The results indicated no significant difference in the structure and hydrophobicity of the molecular surface between the mutant peptide "SLFDASHML" and the wild-type peptide “SLFDVSHML” (Fig. 6g). Similarly, the amino acid substitution resulting from the MTMR6 _p.A599T_ mutation did not significantly modify the structure or surface hydrophobicity in “RYSEYTEEF” (Fig. 6h). The predictions from NetMHCpan indicated that these mutant peptides, which exhibited strong binding, had minimal difference in binding affinity compared to the wild-type peptides (Table 3). This could be explained by the fact that the mutation site does not align with the canonical anchor motif, which is typically involves amino acid at the P2 or PΩ position in peptides. To further examine the binding ability of mutant peptides to HLA-I molecules, we employed peptide-protein docking using HPepDock. The mutant peptide “SLFDASHML” and wild-type peptide “SLFDVSHML” were examined in complex with HLA-A0201, while the mutant peptide “RYSEYTEEF” and wild-type peptide “RYSEYAEEF” were investigated in complex with HLA-A2402(Fig. 6i, j). The results revealed that the binding energy of HLA-I molecule for the mutant peptide was comparable to that of the wild-type peptide, supporting the predicted HLA-I affinity. The mutant peptide and wild-type peptide exhibit slight differences in their position and conformation within the protein. Furthermore, the hydrogen bond between the peptide and protein has undergone alterations. This finding suggests that amino acid alterations resulting from mutations can impact the peptide's ability to bind to HLA-I molecules, indicating that mutations, even if not situated at the P2 or PΩ position, can still influence the affinity of peptides for the HLA-I molecule.

## Discussion

Neoantigen-based immunotherapy, comprised of adoptive cell therapy, tumor vaccines, and bi-specific antibody therapy, holds significant potential for the treatment of cancers [5–7]. The successful identification of tumor-specific mutant peptides is essential for uncovering potential neoantigens. Immune cells have the ability to recognize tumor cells that present immunogenic mutant peptides on cell surface, resulting in engagement with cytotoxic T cells [38, 39]. However, the identification of neoantigens remains a challenge. Regardless of whether it is based on genomics or whole cell genomic-proteomics, there are several limitations and shortcomings [12, 17]. The low identification efficiency and uncertain immunogenicity significantly restrict the clinical application of neoantigen-based therapy. The discovery of mutant peptides from the HLA immunopeptidome holds significant importance. Recent studies have begun to investigate the HLA immunopeptidome, and emerging evidence has shown successful identification of neoantigens in melanoma, lung cancer, glioblastoma and breast cancer [12, 40–42]. However, this method is still relying on genomic-proteomic identification and the utilization of genomic databases for the discovery of mutant peptides.

Previous studies have suggested that neoantigens are more likely to be discovered in tumors with high TMB [19, 43, 44]. Moreover, low TMB cancers typically exhibit decreased responsiveness to current first-line therapies, such as immune checkpoint therapy and targeted therapy [20]. This further emphasizes the importance of neoantigens. HCC is characterized by a low-to-medium TMB, indicating a relatively low mutation frequency (less than 5 mutations per Mb gene). However, the combination of low mutation frequency, inadequate sequencing depth in WES, and high level of heterogeneity poses challenges in identifying neoantigens in HCC. In a study involving 16 HCC patients, only 11 neoantigens were identified from a total of 1,039 non-synonymous mutations. Notably, no neoantigens were detected in HLA immunopeptides [19], highlighting the infrequent presentation of mutant peptides by HLA complexes in HCC. This finding underscores the difficulties associated with neoantigens identification in HCC. Due to the challenges in obtaining clinical samples from patients with advanced HCC, we initially described the HLA-I immunopeptidome of the HepG2 cell line. This dataset serves as a valuable reference for future studies involving other cell lines and tissue samples from HCC.

In this study, we initially characterized the HLA-I immunopeptidomes of HepG2 cell line using immunoprecipitation. Although the western blot analysis revealed an enrichment efficiency of approximately 50% compared to TCL. Previous research has demonstrated that HLA-I immunopeptides can be completely eluted from HLA-I molecules at a pH of 3.3 [45, 46]. In this manuscript, the elution buffer, primarily consisting of 0.15% TFA, was adjusted to a pH of approximately 2.0 to ensure the elution of the majority of peptides for subsequent MS analysis. Surprisingly, one mutant peptide was identified, indicating a relatively low efficiency in discovering neoantigens using the HepG2 WES-based database. Comparable outcomes have been observed in previous studies as well. For instance, lung adenocarcinoma cell lines H1975 and PC9 detected only 3 and 4 mutant peptides, respectively; Moreover, 5 patient-derived organoids from colorectal cancers revealed the presence of 3 potential neoantigens; and even in high TMB cancers like melanoma, 5 potential neoantigens were identified using a criterion of FDR = 1% [16, 47]. These studies imply that personal WES or genomic-based database can be used for identifying neoantigens, despite their relatively low efficiency. Efforts have been made to address this issue. For example, immune peptide databases that compile immunopeptidome information from global research have been established, such as the Immune Epitope Database (IEDB), TSNAdb, and databasePepNeo [48–50]. These databases provide sequences of mutant peptides, information about HLA-I affinity, and experimental evidence of immunogenicity. However, neoantigen databases primarily serve to verify identified peptides rather than discover mutant peptides from MS data files.

We generated a COSMIC based HCC somatic mutation, which includes the most comprehensive collection. This database was constructed using mutation genes obtained from COSMIC, widely acknowledged as a detailed resource for cancer somatic mutations and extensively utilized in various cancer research studies. A similar strategy was employed to enhance the identification of non-canonical peptides. This was achieved by generating an ENSEMBL-based proteogenomics database, which unveiled that non-canonical peptide constituted over 5% of the total number of identified peptides in 65 cell line datasets [51]. However, databases generated from filtered non-synonymous mutations tend to be large, significantly impacting search efficiency and compatibility with certain software. To address this, we compressed the database by retaining only the upstream and downstream amino acid sequences surrounding the mutation site. By employing this strategy, we successfully generated a COSMIC-based database that is less than 50 Mb in size. This compressed database can be utilized with the majority of proteomics analysis software. The main advantage of the COSMIC-based database is its ability to encompass a wide range of mutations, including low frequency mutations, which potentially uncovers a greater number of mutant peptides compared to HepG2 WES-based database. This study presents high-quality HepG2 HLA-I immunopeptidomes for the evaluation of COSMIC-based database. Our results demonstrate that the COSMIC-based database successfully identifies a higher number of mutant peptides without the reliance on prior WES sequence data. Further exploration revealed its ability to discover immunopeptides with high spectral quality using COSMIC-based database. However, it is important to note that some peptides identified in the COSMIC-based database exhibited relatively low quality, potentially leading to the misidentification of wild-type peptides as mutant peptides. To mitigate this issue, manual inspection is necessary to remove peptides with poor spectral quality. Overall, adopting this strategy improves the identification of mutant peptides from HLA-I immunopeptidomes without a significant increase in the false positive rate.

To confirm the potential of the COSMIC-based database as a resource for mutant peptides, we evaluated the immunoaffinity of these peptides to assess the feasibility of employing the COSMIC-based database for neoantigen identification. These mutant peptides, characterized by high spectral quality, also demonstrated strong HLA-I affinity according to in silico prediction. Within the COSMIC-based database, MTMR6 _p.A599T_ and TP53 _p.P72R_ were also found in other cancers, indicating the occurrence tumorigenic signaling pathways shared among various cancers. TP53 _p.P72R_ was observed at a frequency ranging from 0.4 to 0.7 in all ethnic groups, and its association with an increased risk of cancer remains controversial [52]. Additionally, two mutant peptides were validated in the IEDB, confirming their presentation by tumors and their affinity for HLA-I molecules. Through peptide-protein docking modeling, it was revealed that these peptides exhibited lower binding energy to HLA-I molecules, indicating a higher likelihood of binding. HLA-I molecules bind antigen peptide through a groove structure, with a typical binding to the second and last amino acid of antigen peptides [53, 54]. The anchor residues Leu/Met and Leu/Val are commonly observed in immunopeptides binding to HLA-A0201. Both the mutant peptide "SLFDASHML" and the corresponding wild-type peptide "SLFDVSHML" fulfill this requirement. Moreover, the mutant peptides "RYSEYTEEF" meet the binding criteria for HLA-A2402. It is important to noted that substitutions of amino acids resulting from mutations at positions other than P2 or PΩ might have a limited impact on the binding process. Indeed, previous studies have confirmed that neoantigens can arise not only from coding genes, but also from non-coding region or even microorganism [55, 56]. Mutant peptides originating from these sources identified from HLA immunopeptidome are still undergoing investigation.

In summary, we present an analysis of the characteristics of HepG2 HLA-I immunopeptidome. To enhance the efficiency of neoantigen identification from the HLA-I immunopeptidome, we developed an HCC COSMIC-based mutation database. Our results suggest that the COSMIC-based database demonstrates superior effectiveness in identifying tumor-specific mutant peptides and neoantigens compared to the HepG2 WES-based database. This strategy allows us with a broader range of potential neoantigen targets for precision immunotherapy.

### Supplementary Information

**Additional file 1**: **Figure S1. A **Whole picture of HLA-A/B western blot. **B. **Scatter plot experimental RT and predicted RT for PSM from 3 biological replicates of HepG2 cell line. **C.** Overlap of 9 a.a identified peptides for three biological replicates from HepG2 cells using Uniprot Human database.** Figure S2. A** Log_2_ intensity for binding peptides predicted by NetMHCpan, ranked by peptide length. **B** HLA-I immunopeptides main binding motifs by Gibbs cluster, when cluster number = 1. **C** HLA-I immunopeptides main binding motifs by Gibbs cluster, when cluster number = 3. **D** Scatter plot shows the proportion of P2 and P3 amino acids in the Gibbs clustered peptides and NetMHCpan HLA-A0201 data set. **E** Scatter plot shows the proportion of P2 and P3 amino acids in the Gibbs clustered peptides and NetMHCpan HLA-A2402 data set.** Figure S3. A **Tumor tissue distribution of COSMIC-reported somatic mutations in the identified mutant peptides using COSMIC-based database. **B **The proportion of equal weight peptides to unique peptides HepG2 WES-based or COSMIC-based database.** Figure S4. A** MS2 spectrum of identified binder mutant peptides using COSMIC-based database. **B** MS2 spectrum of identified non-binder mutant peptides using COSMIC-based database.** Table S1.** HLA-I peptidome identification from UniProt Human database

**Additional file 2**: List of HepG2 HLA-I class I peptides from MSFragger search.

**Additional file 3**: Observed and predicted retention time of HLA-I class I peptides.

**Additional file 4**: HepG2 mutation identified by whole exsome sequencing.

**Additional file 5**: Detailed data of HLA-I class I peptides Gibbs cluster.

**Additional file 6**: COSMIC mutation generated from COSMIC database.

**Additional file 7**: Results of MSFragger search using HepG2 WES-based and COSMIC-based database.

**Additional file 8**: Detailed data of peptides binding affinity to HLA-I molecules.

## Data Availability

The raw MS-based sequencing files of HepG2 HLA-I immunopeptidome have been deposited to iProX Integrated Proteome Resources with identifier IPX0007010000, ProteomeXchange identifier PXD045203. The transcriptomic data have been published in our previous work [32].
