# Correction to: COSMIC‑based mutation database enhances identification efficiency of HLA‑I immunopeptidome

**DOI:** 10.1186/s12967-024-05187-7

**Published:** 2024-04-22

**Authors:** Fangzhou Wang, Zhenpeng Zhang, Mingsong Mao, Yudai Yang, Ping Xu, Shichun Lu

**Affiliations:** 1 Medical School of Chinese People’s Liberation Army (PLA), Faculty of Hepato-Pancreato-Biliary Surgery, Institute of Hepatobiliary Surgery of Chinese PLA, Key Laboratory of Digital Hepatobiliary Surgery PLA, Chinese PLA General Hospital, 28 Fuxing Road, Haidian District, 100853 Beijing, China; 2 State Key Laboratory of Proteomics, National Center for Protein Sciences (Beijing), Beijing Proteome Research Center, Institute of Lifeomics, Research Unit of Proteomics and Research and Development of New Drug of Chinese Academy of Medical Sciences, 38 Life Science Park Road, Changping District, 102206 Beijing, China; 3 School of Basic Medical Sciences, Anhui Medical University, Hefei, China; 4 Institute of Medicinal Biotechnology, Chinese Academy of Medical Sciences & Peking Union Medical College, Beijing, China; 5 School of Medicine, Guizhou University, Guiyang, China

Correction to: Journal of Translational Medicine (2024) 22:144

10.1186/s12967-023-04821-0

Following publication of the original article [1], we have been notified that one of the authors’ last name was published incorrectly.

It is now as follows:

Fanghzou Wang1†

It should be as follows:

Fangzhou Wang 1†

The original article was updated.

## References

[1] Wang et al. (2024) COSMIC–based mutation database enhances identification efficiency of HLA–I immunopeptidome (2024). 22:144 10.1186/s12967-023-04821-0. 10.1186/s12967-023-04821-0 PMC10858511 38336780

