# Supplementary material for: COSMIC-based mutation database enhances identification efficiency of HLA-I immunopeptidome
Source: J Transl Med. 2024 Feb 10;22:144. doi: 10.1186/s12967-023-04821-0 (PMC10858511; doi:10.1186/s12967-023-04821-0)
Supplement: Supplementary file 1 — Additional file 1: Figure S1. A Whole picture of HLA-A/B western blot. B. Scatter plot experimental RT and predicted RT for PSM from 3 biological replicates of HepG2 cell line. C. Overlap of 9 a.a identified peptides for three biological replicates from HepG2 cells using Uniprot Human database. Figure S2. A Log2 intensity for binding peptides predicted by NetMHCpan, ranked by peptide length. B HLA-I immunopeptides main binding motifs by Gibbs cluster, when cluster number = 1. C HLA-I immunopeptides main binding motifs by Gibbs cluster, when cluster number = 3. D Scatter plot shows the proportion of P2 and P3 amino acids in the Gibbs clustered peptides and NetMHCpan HLA-A0201 data set. E Scatter plot shows the proportion of P2 and P3 amino acids in the Gibbs clustered peptides and NetMHCpan HLA-A2402 data set. Figure S3. A Tumor tissue distribution of COSMIC-reported somatic mutations in the identified mutant peptides using COSMIC-based database. B The proportion of equal weight peptides to unique peptides HepG2 WES-based or COSMIC-based database. Figure S4. A MS2 spectrum of identified binder mutant peptides using COSMIC-based database. B MS2 spectrum of identified non-binder mutant peptides using COSMIC-based database. Table S1. HLA-I peptidome identification from UniProt Human database [file 12967_2023_4821_MOESM1_ESM.docx]

**COSMIC-based mutation database enhances identification efficiency of HLA-I immunopeptidome**

Supplementary Materials

**Supplementary Figures 1**


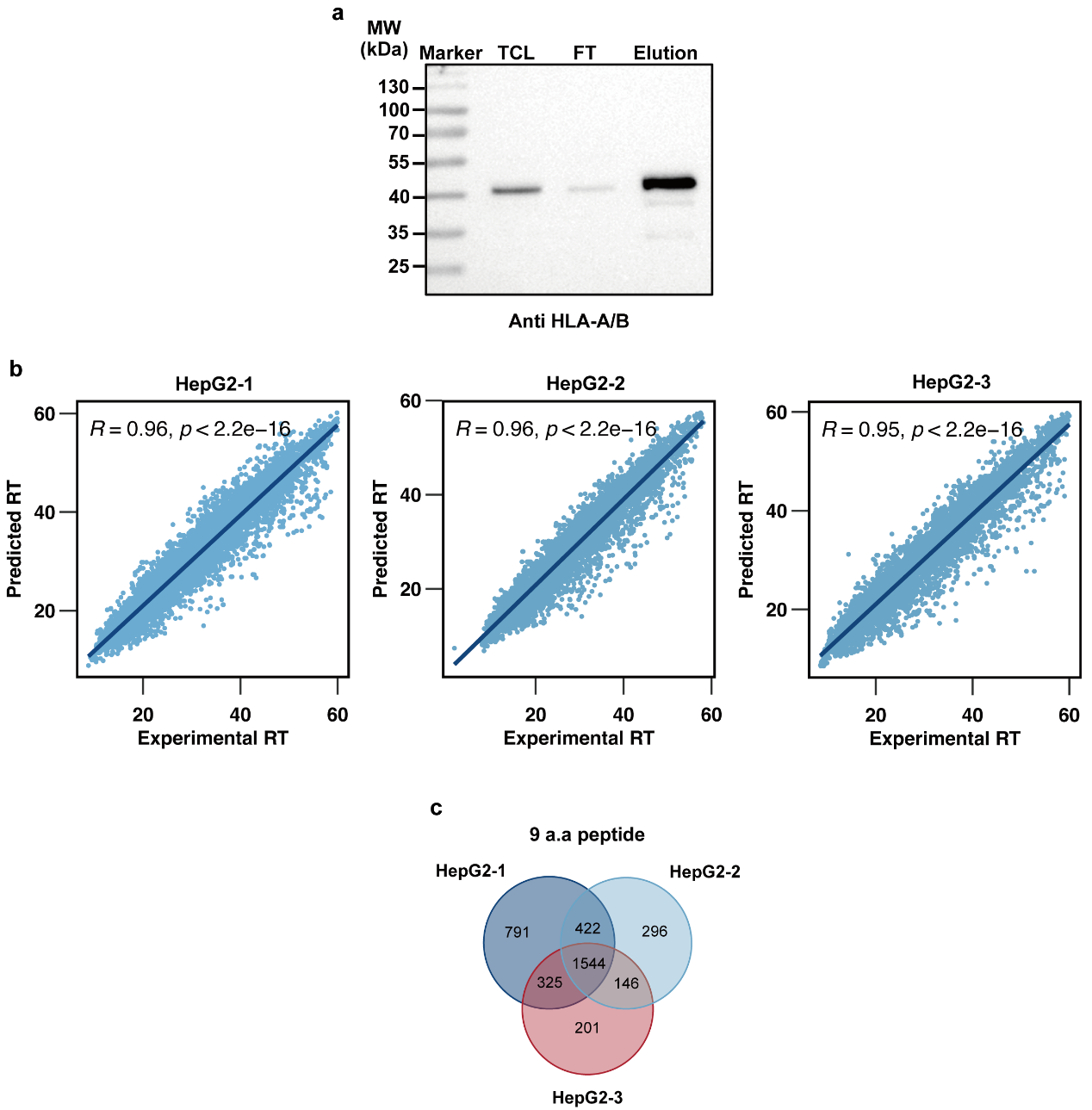


**Supplementary figure. 1**

**A.** Whole picture of HLA-A/B western blot.

**B.** Scatter plot experimental RT and predicted RT for PSM from 3 biological replicates of HepG2 cell line.

**C.** Overlap of 9 a.a identified peptides for three biological replicates from HepG2 cells using Uniprot Human database.

**Supplementary Figures 2**


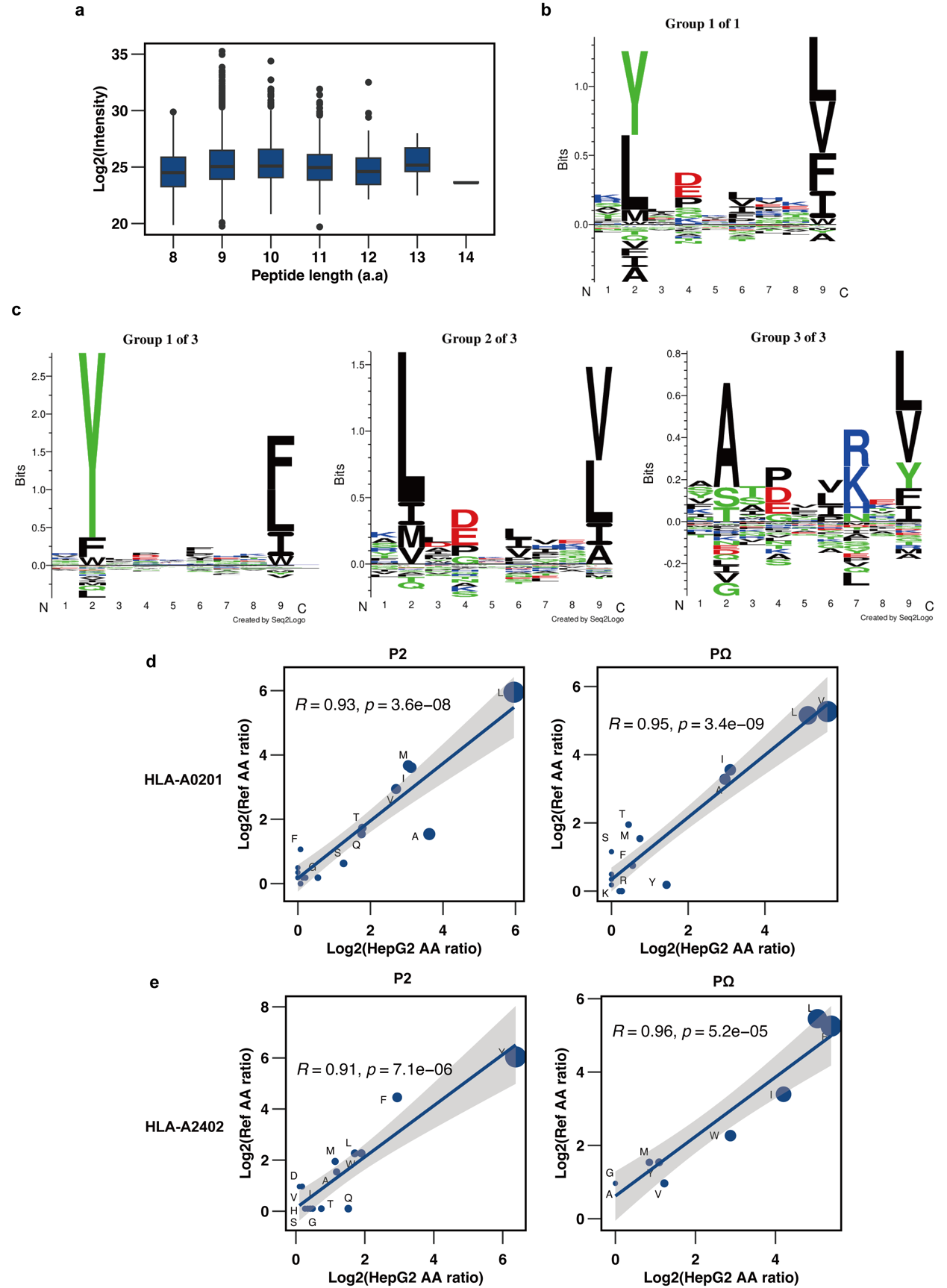


**Supplementary figure. 2**

**A.** Log_2_ intensity for binding peptides predicted by NetMHCpan, ranked by peptide length.

**B.** HLA-I immunopeptides main binding motifs by Gibbs cluster, when cluster number = 1.

**C.** HLA-I immunopeptides main binding motifs by Gibbs cluster, when cluster number = 3.

**D.** Scatter plot shows the proportion of P2 and P3 amino acids in the Gibbs clustered peptides and NetMHCpan HLA-A0201 data set.

**E.** Scatter plot shows the proportion of P2 and P3 amino acids in the Gibbs clustered peptides and NetMHCpan HLA-A2402 data set.

**Supplementary Figures 3**


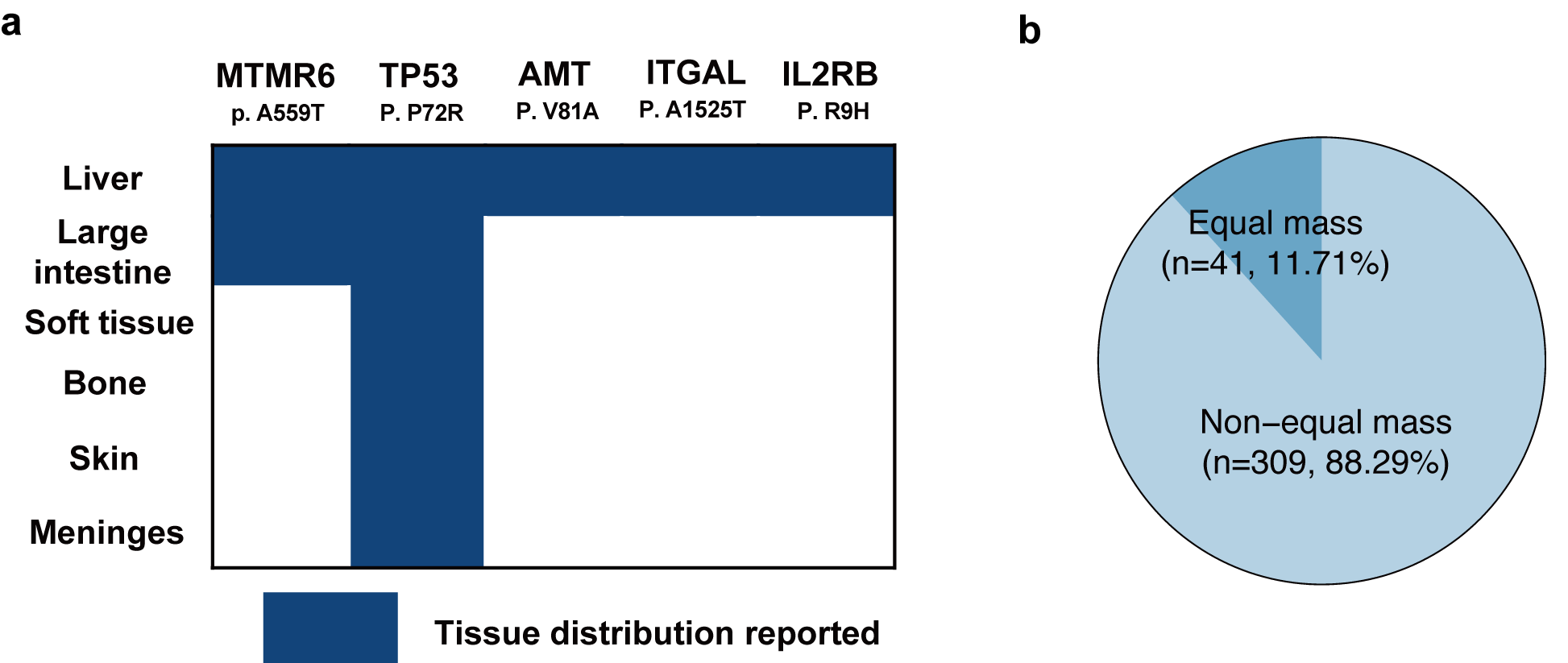


**Supplementary figure. 3**

**A.** Tumor tissue distribution of COSMIC-reported somatic mutations in the identified mutant peptides using COSMIC-based database.

**B.** The proportion of equal weight peptides to unique peptides HepG2 WES-based or COSMIC-based database.

**Supplementary Figures 4**


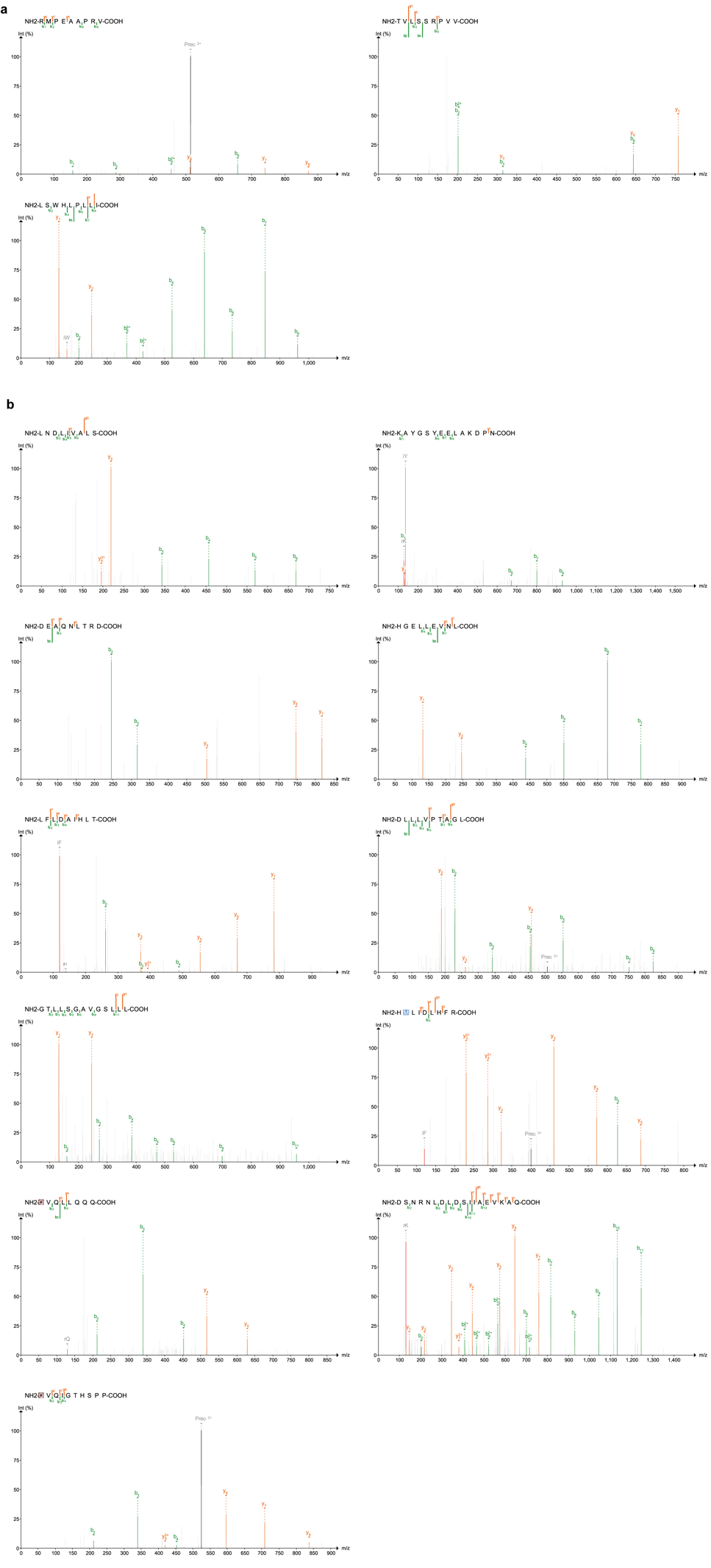


**Supplementary figure. 4**

**A.** MS2 spectrum of identified binder mutant peptides using COSMIC-based database.

**B.** MS2 spectrum of identified non-binder mutant peptides using COSMIC-based database.

**Supplement table 1. HLA-I peptidome identification from UniProt Human database**

| **Sample** | **MS_2_** | **PSM** | **Peptide** | **Protein** | **8-14 a.a**  **Peptide** | **PSM/MS_2_** | **PSM/**  **Peptide** | **Peptide**  **/Protein** |
| --- | --- | --- | --- | --- | --- | --- | --- | --- |
| **HepG2-1** | **51917** | **6497** | **5401** | **2996** | **4537** | **12.5%** | **1.20** | **1.80** |
| **HepG2-2** | **53101** | **5636** | **5004** | **2474** | **3900** | **10.5%** | **1.13** | **2.02** |
| **HepG2-3** | **53079** | **5518** | **4618** | **2193** | **3530** | **10.4%** | **1.19** | **2.52** |
